# Sevoflurane inhibits malignant progression of colorectal cancer via hsa_circ_0000231-mediated miR-622

**DOI:** 10.1186/s40709-021-00145-6

**Published:** 2021-06-28

**Authors:** Jingpeng Wang, Shuyuan Li, Gaofeng Zhang, Huihua Han

**Affiliations:** 1 Department of Anaesthesiology, The Chengyang People’s Hospital, No.76 Zhengyang Road, Chengyang District, Qingdao, 266109 Shandong Province China; 2 Fever Clinic, The Chengyang People’s Hospital, Qingdao, Shandong Province China; 3 Department of Anaesthesiology, The Affiliated Qingdao Municipal Hospital of Qingdao University, Qingdao, Shandong Province China

**Keywords:** Sev, hsa_circ_0000231, miR-622, CRC

## Abstract

**Background:**

Sevoflurane (Sev), a commonly used volatile anesthetic, has been reported to inhibit the process of colorectal cancer (CRC). Circular RNAs (circRNAs) are revealed to participate in the pathogenesis of CRC. This study aims to reveal the mechanism of hsa_circ_0000231 in Sev-mediated CRC progression.

**Methods:**

The expression of hsa_circ_0000231 and microRNA-622 (miR-622) was detected by quantitative real-time polymerase chain reaction (qRT-PCR). Protein level was determined by western blot analysis. Cell proliferation was investigated by 3-(4,5-Dimethylthiazol-2-yl)-2,5-diphenyltetrazolium bromide (MTT), cell colony formation and DNA content quantitation assays. Cell apoptosis was detected by Annexin V-fluorescein isothiocyanate and propidium iodide double staining and caspase 3 activity assays. Cell migration and invasion were investigated by wound-healing and transwell invasion assays, respectively. The putative relationship between hsa_circ_0000231 and miR-622 was predicted by circular RNA Interactome online database, and identified by dual-luciferase reporter and RNA immunoprecipitation assays. The impacts of hsa_circ_0000231 on Sev-mediated tumor formation in vivo were presented by in vivo assay.

**Results:**

Hsa_circ_0000231 expression was upregulated, while miR-622 was downregulated in CRC tissues and cells compared with control groups. Sev treatment decreased hsa_circ_0000231 expression, but increased miR-622 expression in CRC cells. Sev treatment suppressed cell proliferation, migration and invasion, and induced cell apoptosis. Hsa_circ_0000231 overexpression restored Sev-mediated CRC progression in vitro. Additionally, hsa_circ_0000231 acted as a sponge of miR-622, and miR-622 inhibitors reversed the impacts of hsa_circ_0000231 silencing on CRC process. Furthermore, Sev treatment inhibited tumor growth by regulating hsa_circ_0000231 in vivo.

**Conclusion:**

Hsa_circ_0000231 attenuated Sev-aroused repression impacts on CRC development by sponging miR-622. This findings may provide an appropriate anesthetic protocol for CRC sufferers undergoing surgery.

**Supplementary Information:**

The online version contains supplementary material available at 10.1186/s40709-021-00145-6.

## Background

Colorectal cancer (CRC) is a general pernicious cancer worldwide with high mortality [1]. CRC ranks the third in incidence and the second in death rate among various cancers for the combination of men and women [2]. The data studied in 2017 presented nearly half of 1.8 million CRC patients died [3]. At present, despite brilliant achievements have been achieved in disclosing the pathogenesis of CRC, the high mortality of CRC sufferers is still intently correlated with recurrence and metastasis [4, 5]. Major therapeutic manners of CRC are operating together with chemotherapy, radiotherapy or targeted treatment [6]. Numerous studies have verified that Sevoflurane (Sev), a frequently utilized inhaled anesthetic, plays significant part in cancer process. For instances, Liu and his colleagues explained Sev repressed cell growth via increasing microRNA-203 (miR-203) expression in breast cancer [7]. Kang et al. also reported Sev had repressive impacts on cell proliferation and invasion through mediating mitogen-activated protein kinase-related pathway in ovarian cancer [8]. In this paper, the molecular mechanism of CRC development mediated by Sev was revealed.

Circular RNA (circRNA) is a novel noncoding RNA, characterised by high stability, high expression and good conservatism [9]. An increasing number of research efforts have reported that circRNAs are involved in the evolution of various cancers, such as lung carcinoma [10], glioma [11], bladder cancer [12] and CRC [13]. Previous research also presented that circRNAs were related to Sev-mediated cancer process. For example, Li and his colleagues indicated Sev repressed cell proliferation and metastasis by decreasing circ_0002755 and circ_0012129 expression in glioma [14]. He et al. revealed that Sev restrained cell proliferation and induced cell apoptosis via controlling circ-3-hydroxy-3-methylglutaryl-CoA synthase 1 (circ-HMGCS1) in colon cancer [15]. In this study, we found that circ_0000231 (hsa_circ_0000231) was increased in CRC specimen and cell lines, but decreased in Sev-treated CRC cells. Thus, we hypothesized that hsa_circ_0000231 might participate in modulating Sev-mediated CRC progression.

MiRNA is a small RNA with about 20 nucleotides, commonly altering mRNA levels via binding to their non-coding regions [16]. Multiple data exhibited miRNAs regulated cancer progression through interacting with circRNAs, including CRC. For example, enforced circ_100395 expression hindered cell proliferation and metastasis via sponging miR-1228 in lung cancer [10]. Circ_001783 silencing repressed cell proliferation and invasion through binding to miR-200c-3p in breast cancer [17]. In CRC, circ_0026344 modulated cell metastasis, growth and apoptosis via sponging miR-183, miR-21 or miR-31 [18, 19]. In this paper, we found that hsa_circ_0000231 possessed the binding sequence of miR-622, which has been reported to act as a repressor in CRC progression [20].

Herein, the impacts of hsa_circ_0000231 silencing on cell proliferation, metastasis and apoptosis were confirmed. Whether hsa_circ_0000231 was involved in Sev-mediated CRC progression was disclosed by rescue experiments. Additionally, whether hsa_circ_0000231 bound to miR-622 was demonstrated.

## Methods

### Specimen collection and the ethics committee

Forty-seven pairs of human CRC tissues and matched healthy colorectal tissues were collected up from CRC sufferers in the Chengyang People’s Hospital, and the written informed consent was signed by the CRC cases before surgery. Obtained tissues were stored at − 80 °C. The Ethics Committee of the Chengyang People’s Hospital approved this study.

### Cell culture and exposure to Sev

Human CRC cell lines (HCT116 and SW620) and normal human colonic epithelial cell line NCM460 were purchased from Procell (Wuhan, China). HCT116 and NCM460 cells were grown in Roswell Park Memorial Institute-1640 (RPMI-1640; Procell, Wuhan, China), and SW620 cells were cultivated in Leibovitz’s L15 media (L15; Procell, Wuhan, China) at 37 °C in humid condition with 5% CO_2_. Media were supplemented with 10% fetal bovine serum (FBS; Procell, Wuhan, China) and 1% penicillin/streptomycin (Procell, Wuhan, China).

For Sev treatment, HCT116 and SW620 cells were placed in a sealed container with humid atmosphere of 37 °C. Sev (Seebio Biotech, Shanghai) was mixed with 95% air and 5% CO_2_ using a volatilization tank, and gas monitor was used to adjust concentrations of Sev to 1.7%, 3.4% and 5.1%, respectively. Then, the cells were treated with the various concentrations of Sev for 30 min.

### Plasmid construction and oligonucleotide synthesis

The small RNAs against hsa_circ_0000231 (si-hsa_circ_0000231#1, si-hsa_circ_0000231#2 and si-hsa_circ_0000231#3), miR-622 mimics (miR-622), miR-622 inhibitors (anti-miR-622) and control groups (si-NC, NC and anti-NC) were synthesized by GenePharma (Shanghai, China). The overexpression plasmids of hsa_circ_0000231 (hsa_circ_0000231) and control group (circ-NC) were built by Geneseed (Guangzhou, China). Plasmids or oligonucleotides were transfected into cells with TurboFect Reagent (Thermo Fisher, Waltham, MA, USA). The synthesized sequences of oligonucleotides were si-hsa_circ_0000231#1 5′-ACTGAACAGATAAGGGTTTAA-3′, si-hsa_circ_0000231#2 5′-CTGAACAGATAAGGGTTTAAA-3′, si-hsa_circ_0000231#3 5′-CACTGAACAGATAAGGGTTTA-3′, miR-622 5′-ACAGUCUGCUGAGGUUGGAGC-3′, anti-miR-622 5′-GCUCCAACCUCAGCAGACUGU-3′, si-NC 5′-CCTCTACCTGTCGCTGAGCTGTAAT-3′, NC 5′-UUUGUACUACACAAAAGUACUG-3′ and anti-NC 5′-CAGUACUUUUGUGUAGUACAAA-3′.

### Quantitative real-time polymerase chain reaction (qRT-PCR)

A miRNeasy Mini Kit (Qiagen, Valencia, CA, USA) was firstly employed to isolate RNA. After that, cDNA was synthesized with a FastKing RT Kit (Tiangen, Beijing, China) or Qiagen reverse transcription kit (Valencia, CA, USA). Then, SuperReal PreMix Color (Tiangen, Beijing, China) was utilized to detect the content of circRNA/miRNA/mRNA. Obtained data were assessed with the 2^−∆∆Ct^ method with U6 or glyceraldehyde 3-phosphate dehydrogenase (GAPDH) as a reference. The sequences of forward and reverse primers were hsa_circ_0000231 5′-ACTTAGCAGCAGCTCCAC-3′ and 5′-CCACTTCTGTCAGCCATT-3′; miR-622 5′-ACACTCCAGCTGGGACAGTCTGCTGAGGT-3′ and 5′-TGGTGTCGTGGAGTCG-3′; GAPDH 5′-GGTCACCAGGGCTGCTTT-3′ and 5′-GGAAGATGGTGATGGGATT-3′; U6 5′-CTCGCTTCGGCAGCACA-3′ and 5′-AACGCTTCACGAATTTGCGT-3′.

### RNase R treatment assay

Cultured HCT116 and SW620 cells were harvested and RNA was isolated according to the method as described above. Then, obtained RNA was incubated with RNase R (RNase R+) (3 U μg^−1^ RNA; Geneseed, Guangzhou, China) and without RNase R (RNase R-) at 37 °C, respectively. About 30 min later, RNeasy MinElute Cleaning Kit (Qiagen, Valencia, CA, USA) was employed to purify RNA. Hsa_circ_0000231 content was determined by qRT-PCR with linear GAPDH mRNA (linear mRNA) as a reference.

### 3-(4,5-Dimethylthiazol-2-yl)-2,5-diphenyltetrazolium bromide (MTT) assay

Cultivated HCT116 and SW620 cells were diluted in RPMI-1640 (Procell, Wuhan, China) and L15 media (Procell, Wuhan, China), respectively, and grown in 96-well plates. Sixteen hours later, cells were treated with Sev (1.7%, 3.4% or 5.1%) (Sigma, St. Louis, MO, USA), si-hsa_circ_0000231#1, si-hsa_circ_0000231#2, hsa_circ_0000231 or anti-miR-622 based on the defined purposes with 0% Sev (Control), si-NC, circ-NC or anti-NC as a reference. At 48 h after treatment, MTT solution (Beyotime, Shanghai, China) was incubated with cells for 4 h. Then, dimethyl sulfoxide (Sigma, St. Louis, MO, USA) was added into plates to dissolve formazan. Cell viability was determined after samples were analyzed with a microplate reader (Thermo Fisher, Waltham, MA, USA) with a wavelength at 490 nm.

### Cell colony formation assay

HCT116 and SW620 cells diluted in RPMI-1640 (Procell, Wuhan, China) or L15 media (Procell, Wuhan, China) were grown in 6-well plates. After various treatments, cells were continued to be cultured for about 14 days. During cell culture, media were renewed every 3 days. Then, cell supernatant was removed, and paraformaldehyde (Sigma, St. Louis, MO, USA) and crystal violet (Sigma, St. Louis, MO, USA) were dripped into plates, respectively. Cell colony-forming ability was determined by analyzing the number of colonies. A colony was considered when cell numbers over 50.

### DNA content quantitation assay

Cells were collected, and eluted with phosphate buffer solution (PBS; Procell, Wuhan, China). Afterwards, the cells were fixed with 70% ethanol (Millipore, Bradford, MA, USA). RNase A (Solarbio, Beijing, China) was incubated with the cells at 37 °C in water. Cells were stained with propidium iodide (PI; Solarbio, Beijing, China) in dark. Finally, samples were analyzed with a flow cytometry (Thermo Fisher, Waltham, MA, USA).

### Annexin V-fluorescein isothiocyanate (Annexin V-FITC) and PI double staining assay

Cell apoptosis was assessed with an Annexin V-FITC/PI apoptosis detection kit (Solarbio, Beijing, China). In brief, harvested cells were suspended in Binding buffer (Solarbio). Cell supernatant was discarded by centrifuging at 300*g* for 12 min, followed by the incubation with Annexin V-FITC (Solarbio, Beijing, China) and PI (Solarbio, Beijing, China) in dark. Finally, cells were diluted in PBS (Procell, Wuhan, China) and assessed with flow cytometer (Thermo Fisher, Waltham, MA, USA).

### Caspase-3 activity assay

A caspase-3 activity detection kit (Beyotime, Shanghai, China) was employed to detect caspase-3 activity. Briefly, cell supernatant was discarded and cells were collected up by centrifuging. Then, lysis buffer (Beyotime, Shanghai, China) was utilized to lyse cells. Supernatant was harvested by centrifugation. Detection buffer (Beyotime, Shanghai, China) and acetyl-Asp-Glu-Val-Asp p-nitroanilide were mixed with the samples, and the output of wavelength at 405 nm was determined with a microplate reader (Thermo Fisher, Waltham, MA, USA). Finally, caspase-3 activity was determined by assessing the output.

### Wound-healing assay

Cell migration was demonstrated in this part. In short, cells were grown in 6-well plates after diverse treatments. Cell wounds were created when the confluence of cells reached approximately 100%. Cells were cultured in RPMI-1640 (Procell, Wuhan, China) or L15 media (Procell, Wuhan, China) without FBS (Procell, Wuhan, China). After 24 h, the migratory capacity of cells was determined by analyzing the width of wounds under microscope (Nikon, Tokyo, Japan) at a 40× magnification.

### Transwell invasion assay

The invasive ability of CRC cells was revealed with transwell chambers with Matrigel (Corning, Madison, New York, USA). In brief, HCT116 and SW620 cells were cultivated in the upper chambers supplemented with FBS-free RPMI-1640 (Procell, Wuhan, China) and L15 media (Procell, Wuhan, China), respectively. RPMI-1640 (Procell, Wuhan, China) and L15 media (Procell, Wuhan, China) containing 15% FBS (Procell, Wuhan, China) were added into the lower chambers. At 24 h after culture, supernatant was removed, and cells were orderly incubated with paraformaldehyde (Sigma, St. Louis, MO, USA) and crystal violet (Sigma, St. Louis, MO, USA). Results were determined by counting cell numbers in the lower chambers under microscope (Nikon) with a 100× magnification.

### Dual-luciferase reporter assay

Circular RNA Interactome online database (https://circinteractome.nia.nih.gov/api/v2/mirnasearch?circular_rna_query=hsa_circ_0000231&mirna_query=hsa-miR-622&submit=miRNA+Target+Search) was firstly utilized to predict the binding sites of hsa_circ_0000231 in miR-622. Then, the wild-type (wt) and mutant (mut) plasmids of hsa_circ_0000231 were constructed by Geneseed Co., Ltd. (Guangzhou, China) and named as hsa_circ_0000231-wt and hsa_circ_0000231-mut, respectively. Built plasmids were co-transfected into HCT116 and SW620 cells with miR-622 or NC with TurboFect Reagent (Thermo Fisher, Waltham, MA, USA) based on the instruction of manufacturer. Forty-eight hours later, luciferase activities were detected by a Dual-Lucy Assay Kit (Solarbio, Beijing, China). *Renilla* luciferase activity was used as a reference.

### RNA immunoprecipitation (RIP) assay

Cells were lysed with RIP lysis buffer (Millipore, Bradford, MA, USA). Then, the lysates were incubated with magnetic beads coated with the antibodies against argonaute-2 (Anti-Ago2; Abcam, Cambridge, UK) or immunoglobulin G (Anti-IgG; Abcam, Cambridge, UK) for 24 h. Proteins were digested with proteinase K (Millipore, Bradford, MA, USA), and the contents of hsa_circ_0000231 and miR-622 were detected by qRT-PCR.

### In vivo assay

The Vital River Laboratories (Beijing, China) provided male BALB/c nude mice (5 weeks of age), and the mice were fed in a sterile environment. All mice were divided into four groups (n = 5, respectively). SW620 cells were cultivated for 24 h after treatment of 5.1% Sev, and the cells were transfected with hsa_circ_0000231 or circ-NC. After 48 h, the cells were digested and diluted in 200 μL PBS (Procell, Wuhan, China), which were hypodermically inoculated right anterior temporal region of mice. Tumor volume was measured every one day. Five days later, nude mice were sacrificed by intraperitoneal injection of xylazine (10 mg kg^−1^; Seebio Biotech, Shanghai, China) and the neoplasms were harvested. The weight of every tumor was measured. A part of each tumor was kept for further assessment in RNA or protein level. The Animal Care and Use Committee of the Chengyang People's Hospital approved this research.

### Western blot analysis

Tissues were lysed using RIPA buffer (Beyotime, Shanghai, China), and lysates were loaded onto 12% bis–tris-acrylamide gels (Thermo Fisher, Waltham, MA, USA). The separated protein bands were electrotransferred onto polyvinylidene fluoride membranes (Millipore, Bradford, MA, USA), and then immersed in 5% nonfat dry milk (Solarbio, Beijing, China). After that, the membranes were incubated with anti-BCL2-associated x protein (anti-Bax) (1:5000; Abcam, Cambridge, UK), anti-matrix metalloprotein 2 (anti-MMP2) (1:5000; Abcam, Cambridge, UK), anti-MMP9 (1:3000; Abcam, Cambridge, UK), anti-caspase 3 (anti-t-caspase 3) (1:5000; Abcam, Cambridge, UK), anti-cleaved-caspase 3 (anti-C-caspase 3) (1:8000; Abcam, Cambridge, UK), anti-proliferating cell nuclear antigen (anti-PCNA) (1:3000; Abcam, Cambridge, UK), anti-MYC proto-oncogene, bHLH transcription factor (anti-c-Myc; 1:1000; Abcam, Cambridge, UK), anti-KRAS proto-oncogene, GTPase (anti-K-RAS; 1:1000; Abcam, Cambridge, UK), anti-B-Raf proto-oncogene, serine/threonine kinase (anti-BRAF; 1:2000; Abcam, Cambridge, UK) and anti-GAPDH (1:15,000; Abcam, Cambridge, UK), respectively. Then, the membranes were incubated with secondary antibody (1:8000; Abcam, Cambridge, UK). Finally, RapidStep ECL Reagent (Millipore, Bradford, MA, USA) was used to visualize the protein bands. GAPDH was chosen as a control.

### Statistical analysis

Data derived from three independent duplicate tests were assessed by GraphPad Prism (GraphPad Software, La Jolla, CA, USA), and expressed as means ± standard deviations (SD). Significant differences in Spearman correlation analysis and overall survival curve were compared with Spearman’s correlation test and log-rank test, respectively. Additionally, significant differences were compared with two-tailed Student’s *t*-tests or Wilcoxon rank-sum test between the two groups and with one-way analysis of variance (ANOVA) with Tukey’s test or Kruskal–Wallis test between the multiple groups. *p* value < 0.05 was deemed as statistical significance.

## Results

### Hsa_circ_0000231 expression was upregulated in the tissues of CRC patients with poor prognosis

**Fig. 1:**
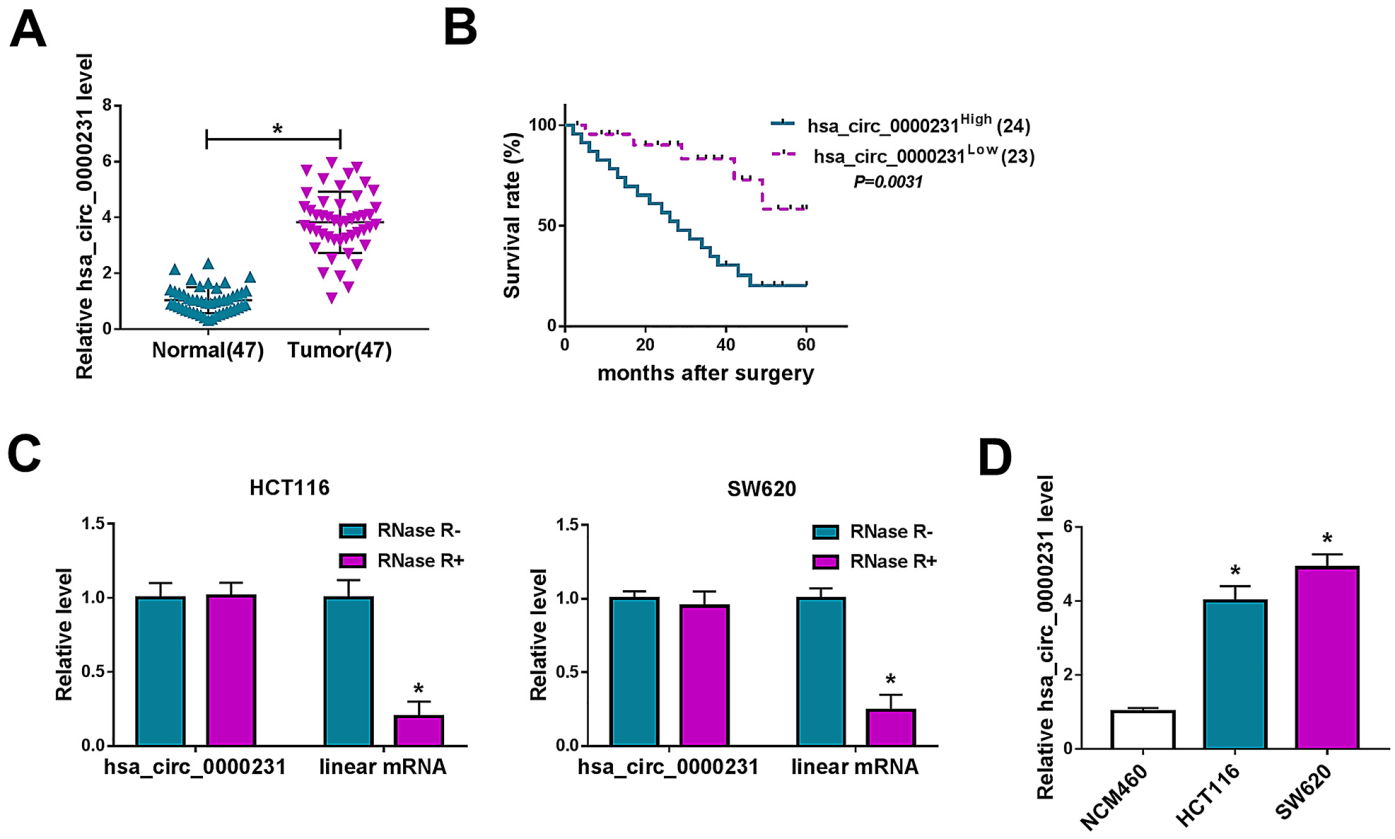
Hsa_circ_0000231 was overexpressed in CRC tissues and cells with poor prognosis of CRC patients. **A**, **D** Hsa_circ_0000231 expression was detected by qRT-PCR in 47 pairs of CRC and matched healthy colorectal tissues as well as NCM460, HCT116 and SW620 cells. **B** The overall survival curve of CRC sufferers with high or low hsa_circ_0000231 expression was assessed by Kaplan–Meier methods. **C** RNase R treatment assay was employed to demonstrate hsa_circ_0000231 was more stable than linear mRNA. *p < 0.05 (Wilcoxon rank-sum test, two-tailed Student’s *t*-tests and ANOVA with Tukey’s test)

Hsa_circ_0000231 expression was firstly determined in CRC tissues, and results showed that its expression was dramatically increased in CRC tissues compared with paracancerous normal colorectal tissues (Fig. 1A). Kaplan–Meier methods presented CRC patients with high hsa_circ_0000231 expression had a low survival rate as compared to those with low hsa_circ_0000231 expression (Fig. 1B), which suggested hsa_circ_0000231 might act as an oncogene in CRC process. Subsequently, RNase R treatment assay showed that hsa_circ_0000231 expression had no obvious change after RNase R treatment, but linear mRNA expression was dramatically decreased (Fig. 1C), suggesting hsa_circ_0000231 was more stable than linear mRNA. Furthermore, the expression level of hsa_circ_0000231 was detected in HCT116 and SW620 cells, and it was found that hsa_circ_0000231 expression was greatly upregulated in HCT116 and SW620 cells relative to NCM460 cells (Fig. 1D). The above data demonstrated hsa_circ_0000231 might positively regulate CRC progression.

### Sev repressed cell proliferation, migration and invasion, whereas induced cell apoptosis in CRC cells

**Fig. 2:**
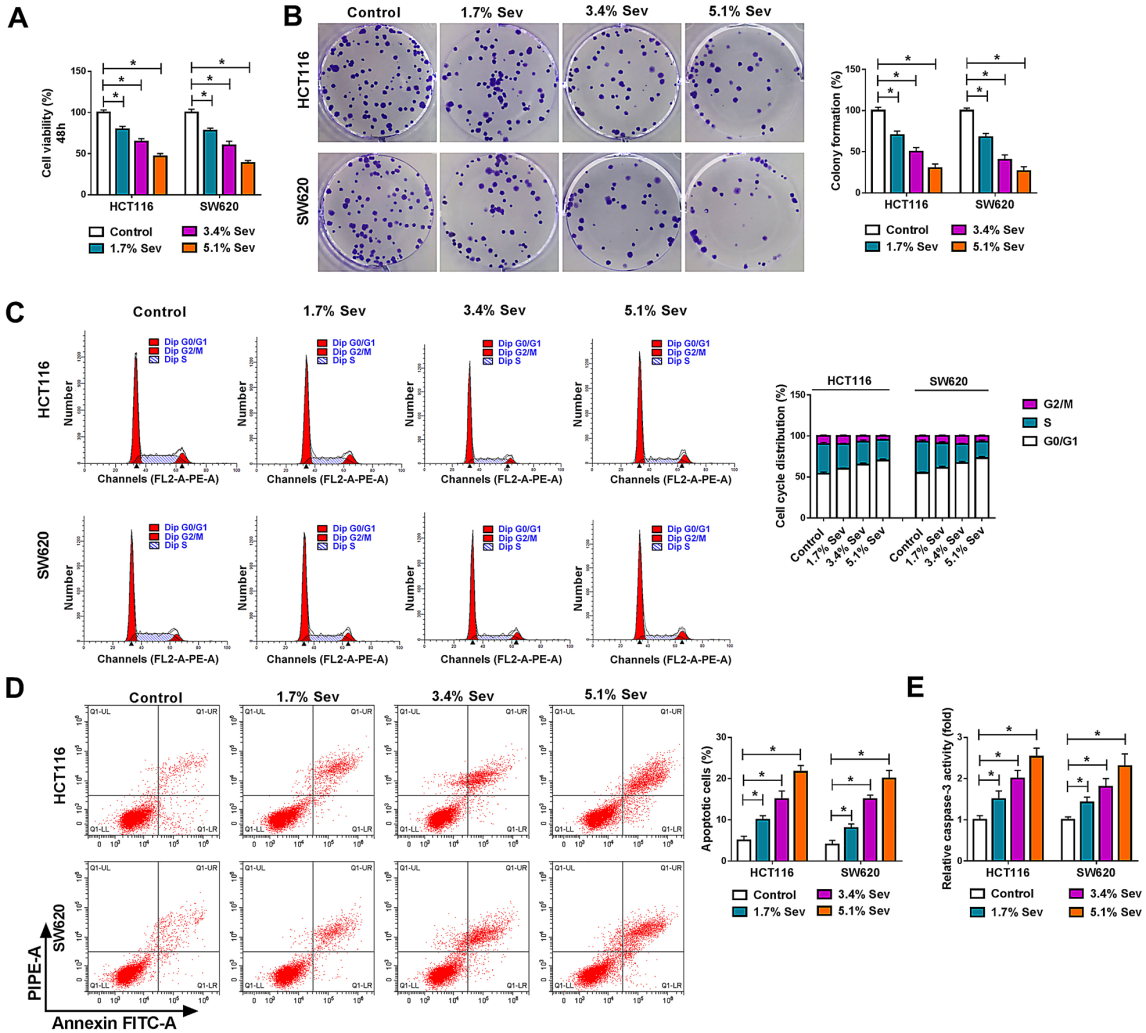
Sev treatment repressed cell proliferation and induced cell apoptosis in CRC. **A,** **B** The impacts of Sev (0%, 1.7%, 3.4% and 5.1%) on cell viability and cell colony-forming ability were presented by MTT and cell colony formation assays, respectively. **C** The effects of Sev (0%, 1.7%, 3.4% and 5.1%) on cell cycle were determined by DNA content quantitation assay in HCT116 and SW620 cells. **D** The effects of Sev (0%, 1.7%, 3.4% and 5.1%) on cell apoptosis were determined by Annexin V-FITC and PI double staining assay in HCT116 and SW620 cells. **E** Caspase-3 activity was determined by caspase-3 activity assay in HCT116 and SW620 cells treated with Sev at various doses (0%, 1.7%, 3.4% and 5.1%). *p < 0.05 (ANOVA with Tukey’s test)

**Fig. 3:**
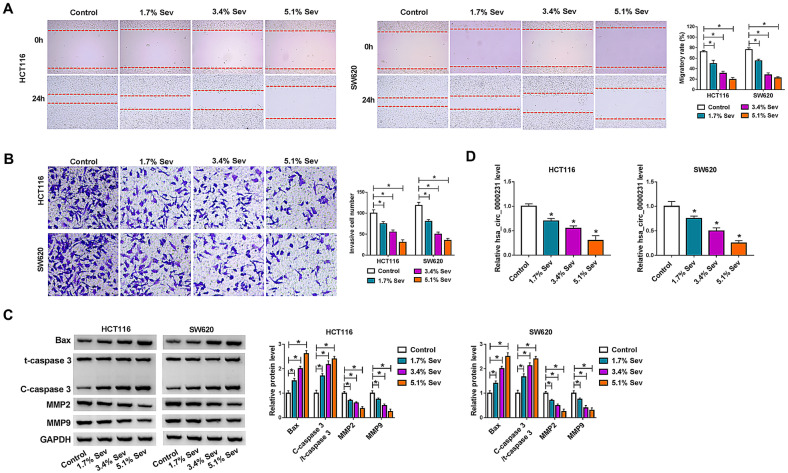
The migration and invasion of HCT116 and SW620 cells were repressed by Sev. **A** The influences of Sev (0%, 1.7%, 3.4% and 5.1%) on the migration of HCT116 and SW620 cells were revealed by wound-healing assay. **B** Transwell invasion assay was employed to demonstrate the impacts of Sev (0%, 1.7%, 3.4% and 5.1%) on the invasion of HCT116 and SW620 cells. **C** The impacts of Sev (0%, 1.7%, 3.4% and 5.1%) on the protein expression of Bax, t-caspase 3, C-caspase 3, MMP2 and MMP9 were determined by western blot analysis in HCT116 and SW620 cells. **D** Hsa_circ_0000231 expression was determined by qRT-PCR in HCT116 and SW620 cells treated with 0%, 1.7%, 3.4% and 5.1% of Sev. *p < 0.05 (ANOVA with Tukey’s test)

The study then explored the impacts of Sev on CRC progression. HCT116 and SW620 cells were initially treated with Sev at various concentrations (1.7%, 3.4% and 5.1%), and cell proliferative and metastatic abilities as well as apoptotic rate were determined. MTT and cell colony formation assays displayed that Sev exposure inhibited cell viability and cell colony-forming ability in a dose-dependent manner (Fig. 2A, B). The cell cycle of HCT116 and SW620 cells was also arrested at G0/G1 phase after Sev treatment in a concentration-dependent manner (Fig. 2C). These findings suggested that Sev treatment repressed the proliferation of HCT116 and SW620 cells. On the contrary, Sev exposure concentration-dependently promoted the apoptosis and caspase-3 activity of HCT116 and SW620 cells (Fig. 2D, E). Additionally, Sev treatment inhibited cell migration and invasion of HCT116 and SW620 cells in dose-dependent fashion (Fig. 3A, B). Sev treatment upregulated Bax protein expression and the value of C-caspase 3/t-caspase 3, and downregulated the protein expression of MMP2 as well as MMP9 in a concentration-dependent manner (Fig. 3C). In support, the protein expression of oncogenes including c-Myc, K-RAS and BRAF was dose-dependently reduced by Sev (Additional file 1: Figure S1A, B). The above data demonstrated Sev could repress CRC process. Given the negative correlation between hsa_circ_0000231 expression and survival rate of CRC sufferers as well as the repressive impacts of Sev on CRC progression, whether Sev regulated hsa_circ_0000231 expression was further analyzed. As shown in Fig. 3D, hsa_circ_0000231 expression was apparently decreased after Sev treatment in a dose-dependent manner, suggesting that Sev might regulate CRC progression by repressing hsa_circ_0000231.

### Hsa_circ_0000231 silencing repressed cell proliferation, migration and invasion, but induced cell apoptosis in HCT116 and SW620 cells

**Fig. 4:**
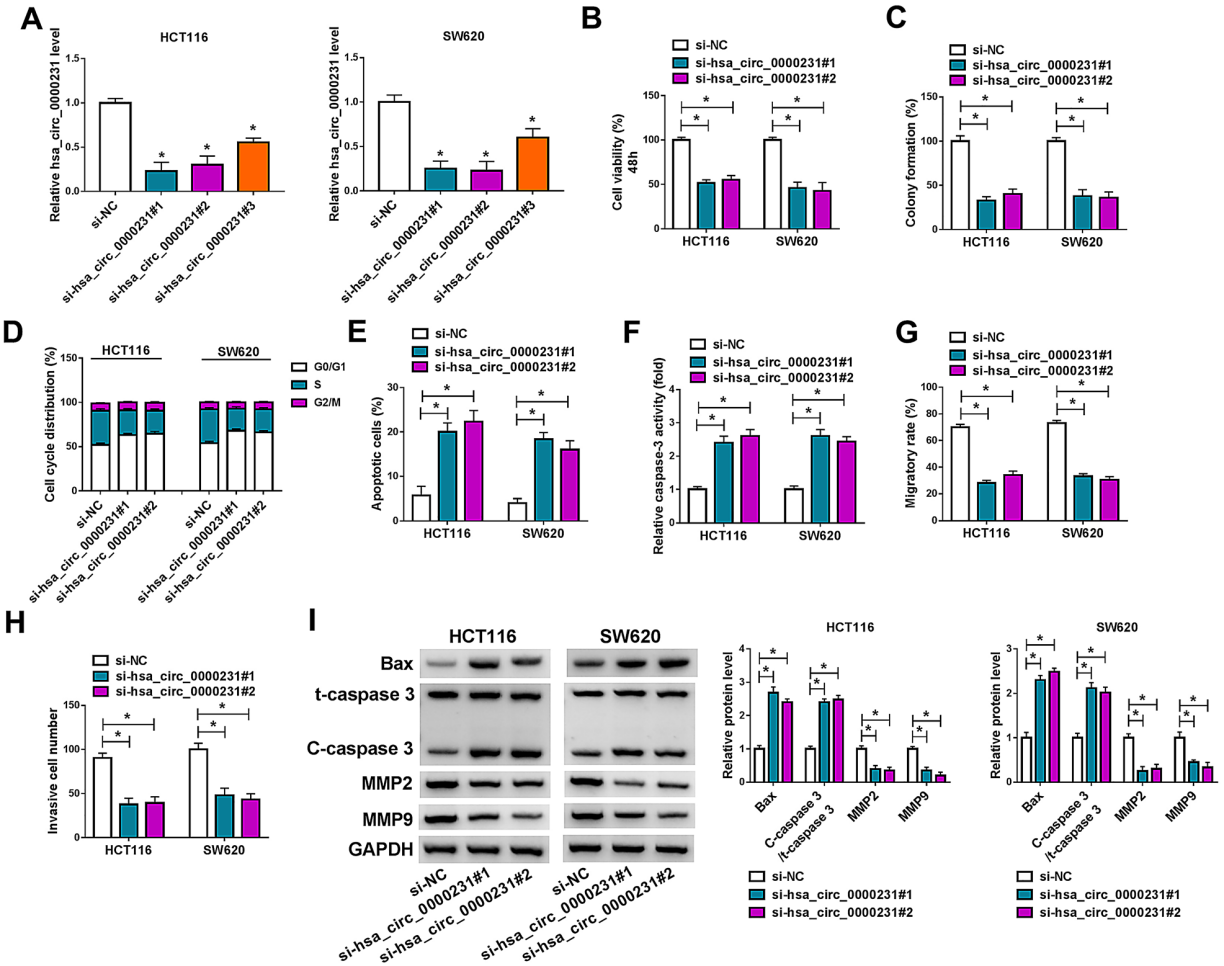
Hsa_circ_0000231 knockdown repressed CRC process. **A** The knockdown efficiency of hsa_circ_0000231#1, si-hsa_circ_0000231#2 and si-hsa_circ_0000231#3 was determined by qRT-PCR in HCT116 and SW620 cells. **B**, **C** The impacts of hsa_circ_0000231 silencing on the viability and colony-forming ability were revealed by MTT and cell colony formation assays, respectively. **D** The effect of hsa_circ_0000231 absence on cell cycle was demonstrated by DNA content quantitation assay. **E** The effect of hsa_circ_0000231 absence on cell apoptosis was demonstrated by Annexin V-FITC and PI double staining assay. **F** The influence of hsa_circ_0000231 silencing on caspase-3 activity was unveiled by caspase-3 activity assay. **G**, **H** Wound-healing and transwell invasion assays were performed to demonstrate the impacts of hsa_circ_0000231 downregulation on the migration and invasion of HCT116 and SW620 cells, respectively. **I** The impacts of hsa_circ_0000231 silencing on the protein expression of Bax, t-caspase 3, C-caspase 3, MMP2 and MMP9 were determined by western blot analysis in HCT116 and SW620 cells. *p < 0.05 (ANOVA with Tukey’s test)

Considering the high hsa_circ_0000231 expression in HCT116 and SW620 cells, the small interfering RNAs against hsa_circ_0000231 were built and their knockdown efficiency was determined. The data from qRT-PCR analysis displayed that hsa_circ_0000231 expression was notably downregulated after transfection of si-hsa_circ_0000231#1, si-hsa_circ_0000231#2 or si-hsa_circ_0000231#3 in HCT116 and SW620 cells (Fig. 4A). si-hsa_circ_0000231#1 and si-hsa_circ_0000231#2 were employed in subsequent study owing to their higher efficiency. Subsequently, our results presented hsa_circ_0000231 silencing suppressed cell viability and colony-forming ability in HCT116 and SW620 cells (Fig. 4B, C). Hsa_circ_0000231 knockdown also induced cell arrested at G0/G1 phase and promoted cell apoptosis (Fig. 4D, E). Meanwhile, the caspase-3 activity was also promoted after hsa_circ_0000231 silencing (Fig. 4F), which further demonstrated that hsa_circ_0000231 downregulation could induce cell apoptosis. The migratory and invasive abilities of HCT116 and SW620 cells were restrained after hsa_circ_0000231 absence (Fig. 4G, H). Hsa_circ_0000231 silencing increased Bax protein expression and the value of C-caspase 3/t-caspase 3, and decreased the protein expression levels of MMP2 as well as MMP9 (Fig. 4I). Thus, these evidences demonstrated that hsa_circ_0000231 positively regulated CRC progression.

### Hsa_circ_0000231 attenuated Sev-mediated effects on CRC process

**Fig. 5:**
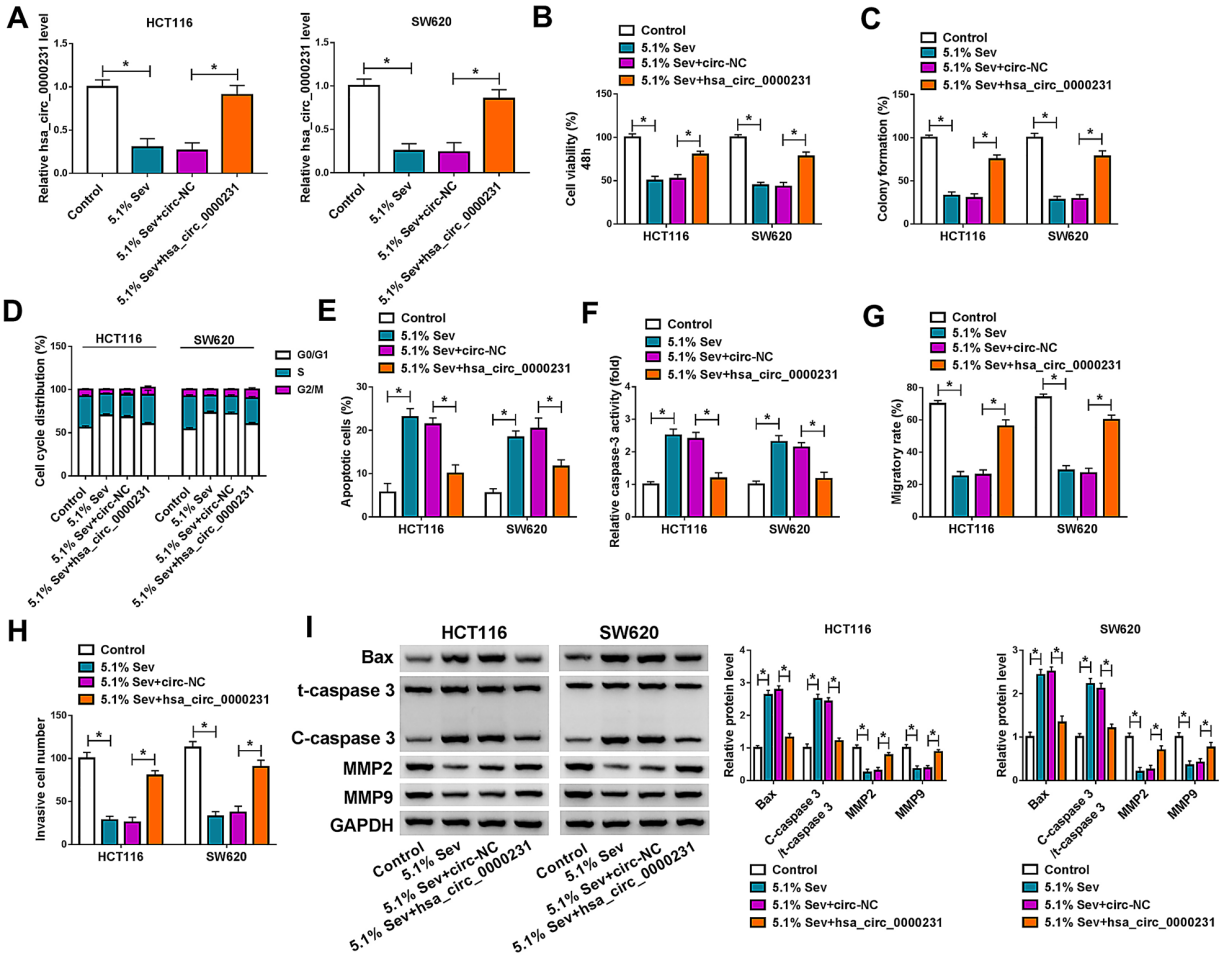
Hsa_circ_0000231 reversed Sev-mediated influences on CRC development. **A** The impacts between Sev treatment and hsa_circ_0000231 overexpression on hsa_circ_0000231 expression were detected by qRT-PCR in HCT116 and SW620 cells. **B**, **C** The impacts between Sev treatment and hsa_circ_0000231 overexpression on the viability and colony-forming ability of HCT116 and SW620 cells were determined by MTT and cell colony formation assays, respectively. **D** DNA content quantitation assay was performed to reveal the effects between Sev treatment and hsa_circ_0000231 overexpression on cell cycle. **E** Annexin V-FITC and PI double staining assay was conducted to show the effects between Sev treatment and hsa_circ_0000231 overexpression on the apoptosis of HCT116 and SW620 cells. **F** Caspase-3 activity assay was used to demonstrate the impacts between Sev treatment and ectopic hsa_circ_0000231 expression on caspase-3 activity. **G**, **H** The impacts between Sev treatment and hsa_circ_0000231 overexpression on the migration and invasion of HCT116 and SW620 cells were revealed by wound-healing and transwell invasion assays, respectively. **I** The effects between Sev treatment and hsa_circ_0000231 overexpression on the protein expression of Bax, t-caspase 3, C-caspase 3, MMP2 and MMP9 were determined by western blot analysis in HCT116 and SW620 cells. *p < 0.05 (ANOVA with Tukey’s test)

Given the repressive role of hsa_circ_0000231 silencing on CRC progression, whether it participated in Sev-mediated CRC process was subsequently explored; the effects between Sev treatment and hsa_circ_0000231 overexpression on CRC progression were analyzed. Results firstly exhibited that hsa_circ_0000231 overexpression reversed the repressive impact of Sev treatment on hsa_circ_0000231 expression in HCT116 and SW620 cells (Fig. 5A). Sev-mediated inhibitory impacts on cell viability and cell colony-forming ability were also restored after hsa_circ_0000231 upregulation (Fig. 5B, C). Additionally, Sev treatment induced cell arrest at G0/G1 phase, whereas ectopic hsa_circ_0000231 expression impaired this impact (Fig. 5D). Sev-induced cell apoptosis was also partly abolished after hsa_circ_0000231 overexpression (Fig. 5E). Caspase-3 activity assay also presented the promotion impact of Sev treatment on caspase-3 activity was hindered after transfection of hsa_circ_0000231 (Fig. 5F). Furthermore, Sev exposure inhibited cell migration and invasion, whereas these impacts were restrained by enforced hsa_circ_0000231 expression (Fig. 5G, H). Sev-mediated effects on the protein expression of Bax, MMP2 and MMP9 as well as the value of C-caspase 3/t-caspase 3, were attenuated after hsa_circ_0000231 overexpression (Fig. 5I). Taken together, the above data suggested that Sev repressed cell proliferation, migration and invasion, and induced cell apoptosis by regulating hsa_circ_0000231.

### Hsa_circ_0000231 acted as a sponge of miR-622

**Fig. 6:**
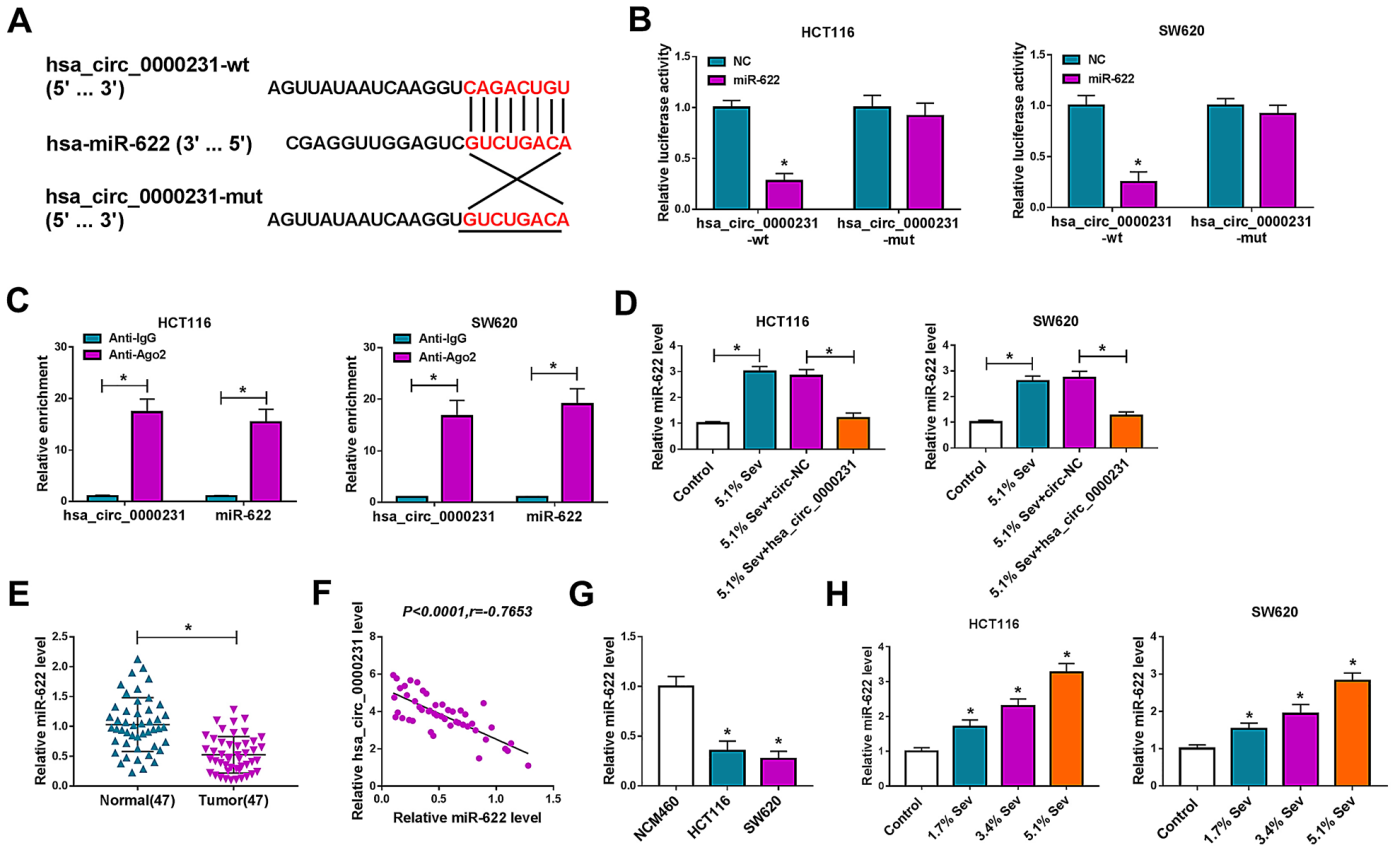
Hsa_circ_0000231 directly interacted with miR-622. **A** Circular RNA Interactome online database was performed to predict the putative relationship between hsa_circ_0000231 and miR-622. **B**, **C** Dual-luciferase reporter and RIP assays were carried out to prove that hsa_circ_0000231 directly bound to miR-622. **D** The impacts between Sev treatment and hsa_circ_0000231 overexpression on miR-622 expression were demonstrated by qRT-PCR in HCT116 and SW620 cells. **E,** **G** MiR-622 expression was detected by qRT-PCR in 47 pairs of CRC and paracancerous normal colorectal tissues as well as NCM460, HCT116 and SW620 cells. **F** Spearman correlation analysis was conducted to disclose the linear relationship between hsa_circ_0000231 and miR-622 expression in CRC tissues. **H** The influences of various concentrations of Sev (0%, 1.7%, 3.4% and 5.1%) on miR-622 expression were determined by qRT-PCR in HCT116 and SW620 cells. *p < 0.05 (two-tailed Student’s *t*-tests, Wilcoxon rank-sum test, ANOVA with Tukey’s test and Spearman’s correlation test)

In order to reveal the regulatory mechanism of hsa_circ_0000231 in CRC process, the miRNA with the ability to bind to hsa_circ_0000231 was sought. Circular RNA Interactome online database presented hsa_circ_0000231 contained the binding sequence of miR-622 (Fig. 6A). To prove the binding relationship between hsa_circ_0000231 and miR-622, dual-luciferase reporter and RIP assays were employed. Results exhibited the relative luciferase activity was dramatically repressed after co-transfection of hsa_circ_0000231-wt and miR-622 in HCT116 and SW620 cells, whereas that had no apparent change in hsa_circ_0000231-mut and miR-622 group (Fig. 6B). RIP assay also presented that both hsa_circ_0000231 and miR-622 were notably enriched by Anti-Ago2 as compared to Anti-IgG in HCT116 and SW620 cells (Fig. 6C). Subsequently, qRT-PCR data revealed miR-622 expression was dramatically upregulated after Sev treatment, but this impact was reversed by enforced hsa_circ_0000231 expression (Fig. 6D). Our results also showed that miR-622 expression was apparently downregulated in CRC tissues and HCT116 and SW620 cells when compared with normal colorectal tissues and NCM460 cells, respectively (Fig. 6E, G). Additionally, a negative correlation between hsa_circ_0000231 expression and miR-622 expression was observed (Fig. 6F). Furthermore, the impact of Sev exposure on miR-622 expression was unveiled in HCT116 and SW620 cells, and we found that miR-622 expression was dose-dependently increased by Sev (Fig. 6H). Thus, our evidences demonstrated hsa_circ_0000231 was directly associated with miR-622, and Sev could upregulate miR-622 expression by regulating hsa_circ_0000231.

### Hsa_circ_0000231 regulated cell proliferation, apoptosis, migration and invasion via binding to miR-622

**Fig. 7:**
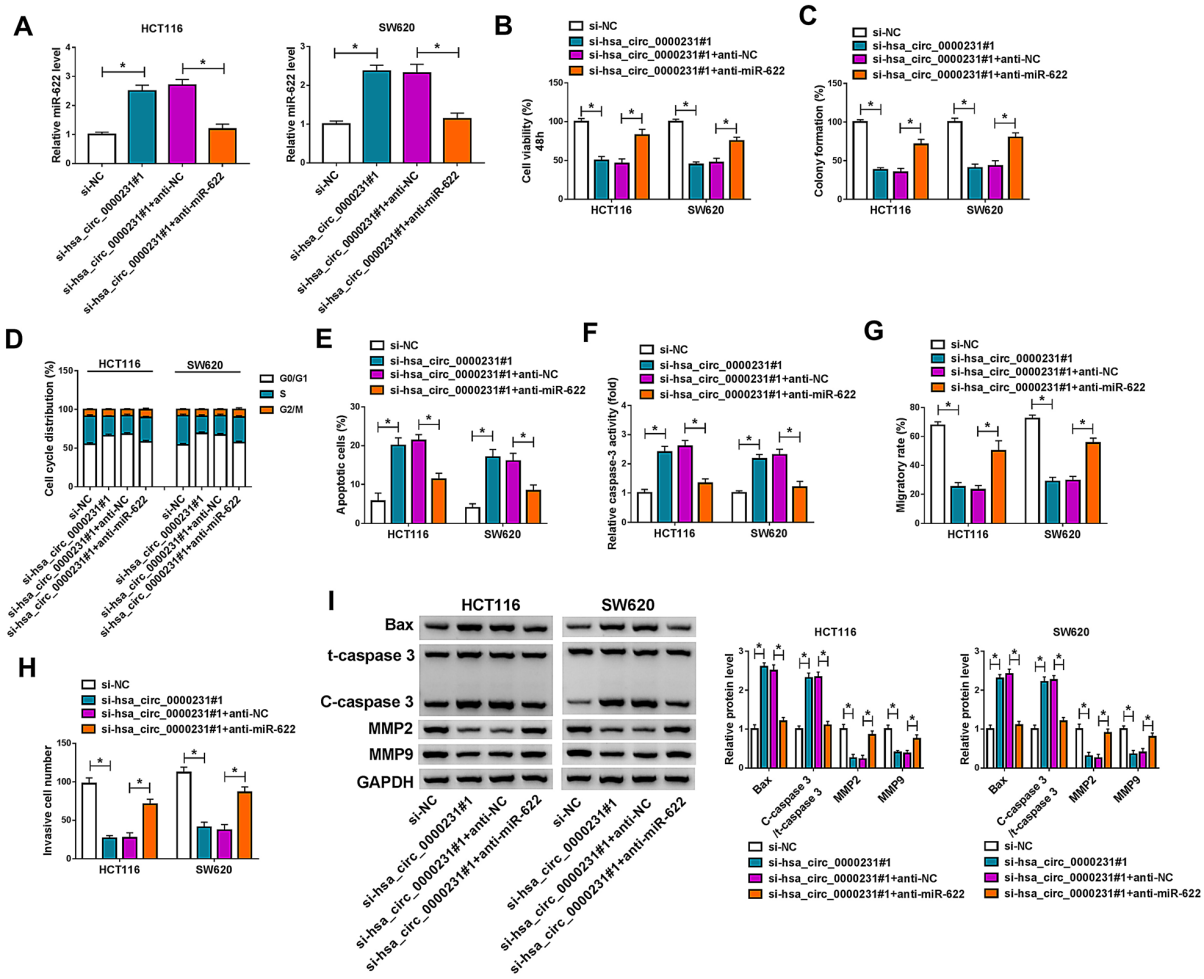
Hsa_circ_0000231 mediated CRC development by binding to miR-622. HCT116 and SW620 cells were transfected with si-NC, si-hsa_circ_0000231#1, si-hsa_circ_0000231#1 + anti-NC and si-hsa_circ_0000231#1 + anti-miR-622, respectively. **A** MiR-622 expression was detected by qRT-PCR in HCT116 and SW620 cells. **B**, **C** The viability and colony-forming ability of HCT116 and SW620 cells were detected by MTT and colony-forming assays, respectively. **D**, **E** Cell cycle and apoptosis were detected by DNA content quantitation assay and Annexin V-FITC and PI double staining assay, respectively, in HCT116 and SW620 cells. **F** Caspase-3 activity was detected by caspase-3 activity assay in HCT116 and SW620 cells. **G**, **H** The migration and invasion of HCT116 and SW620 cells were detected by wound-healing and transwell invasion assays, respectively. **I** Western blot analysis was performed to reveal the influences between hsa_circ_0000231 silencing and miR-622 downregulation on the protein expression of Bax, t-caspase 3, C-caspase 3, MMP2 and MMP9 in HCT116 and SW620 cells. *p < 0.05 (ANOVA with Tukey’s test)

Given the bound relationship between hsa_circ_0000231 and miR-622, whether hsa_circ_0000231 regulated CRC progression by interacting with miR-622 was investigated. Our data firstly showed that hsa_circ_0000231 silencing apparently upregulated miR-622 expression, whereas miR-622 inhibitors attenuated this impact (Fig. 7A). Subsequently, hsa_circ_0000231 knockdown repressed cell viability and cell colony-forming ability, which were reversed after downregulation of miR-622 (Fig. 7B, C). Data also presented that hsa_circ_0000231 silencing induced cell arrest at G0/G1 phase and cell apoptosis, but these effects were restored after transfection of anti-miR-622 (Fig. 7D, E). In order to further analyze the impacts between hsa_circ_0000231 and miR-622 on cell apoptosis, caspase-3 activity assay was performed. Results exhibited that hsa_circ_0000231 downregulation promoted caspase-3 activity, while miR-622 inhibitors restrained this impact (Fig. 7F). Additionally, the migration and invasion of HCT116 and SW620 cells were also repressed by si-hsa_circ_0000231#1; however, these influences were restored after miR-622 downregulation (Fig. 7G, H). The effects of hsa_circ_0000231 downregulation on the protein expression of Bax, MMP2 and MMP9 as well as the value of C-caspase 3/t-caspase 3 were attenuated after transfection of anti-miR-622 (Fig. 7I). Collectively, the above data demonstrated that hsa_circ_0000231 could modulate CRC progression by interacting with miR-622.

### Hsa_circ_0000231 overexpression restrained Sev-mediated impacts on tumor formation in vivo

**Fig. 8:**
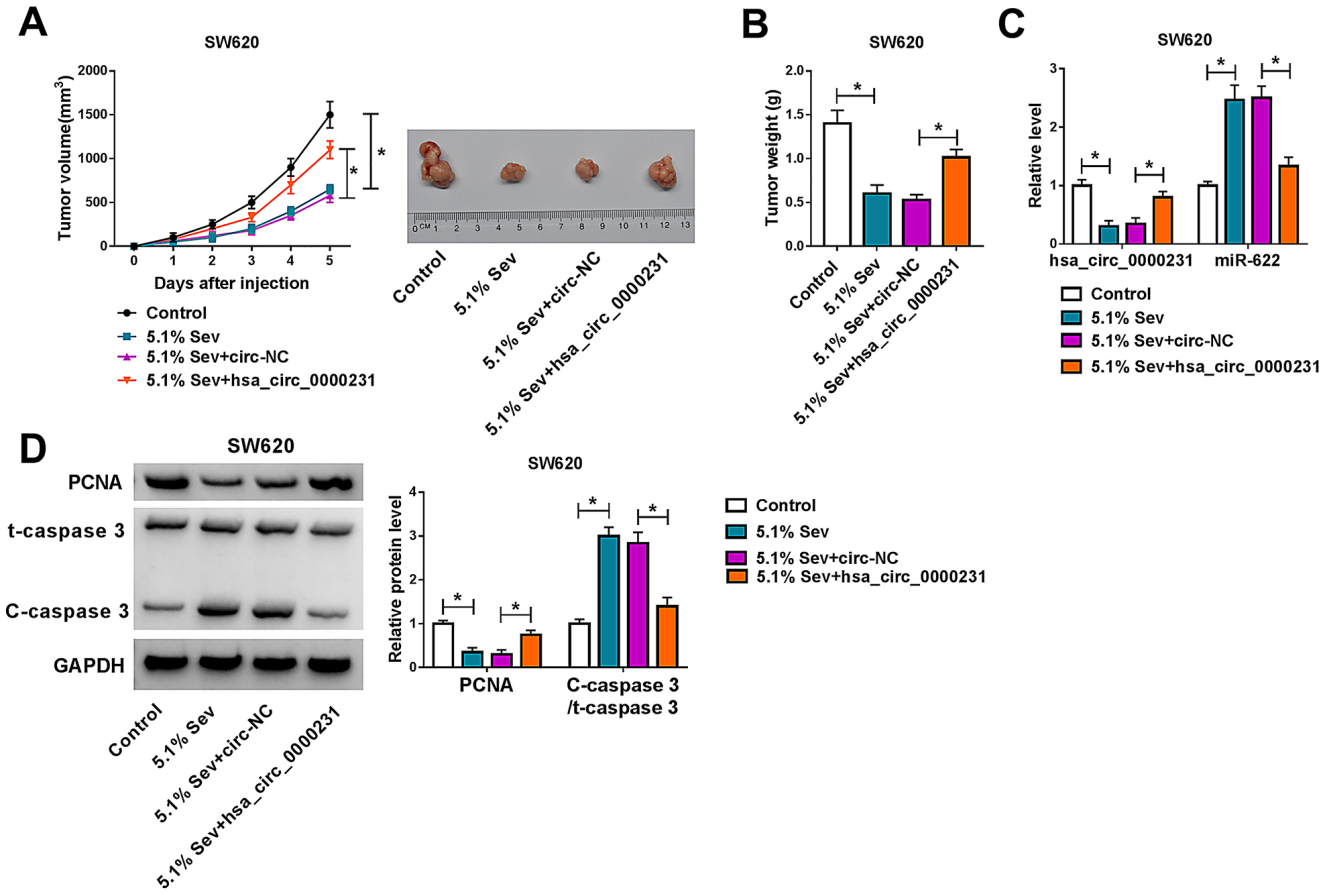
Sev treatment repressed tumor formation by repressing hsa_circ_0000231 in vivo. **A**, **B** The impacts between Sev and hsa_circ_0000231 overexpression on tumor volume and weight were revealed. **C** The effects between Sev treatment and enforced hsa_circ_0000231 expression on the levels of hsa_circ_0000231 and miR-622 were determined by qRT-PCR. **D** Western blot analysis was employed to reveal the influences between Sev treatment and ectopic hsa_circ_0000231 expression on the expression of PCNA protein and the value of C-caspase 3/t-caspase 3. *p < 0.05 (ANOVA with Tukey’s test)

To further affirm the promotion influences of hsa_circ_0000231 on Sev-mediated CRC process, in vivo assay was conducted. Results showed Sev treatment reduced tumor volume and weight, whereas these effects were reversed by hsa_circ_0000231 overexpression (Fig. 8A, B). Additionally, we found hsa_circ_0000231 expression was obviously downregulated, while miR-622 expression was dramatically upregulated after Sev treatment in the excised tissues; however, these impacts were impaired by enforced hsa_circ_0000231 expression (Fig. 8C). Furthermore, the protein expression of proliferation-related maker PCNA was decreased after Sev treatment, which was reversed after hsa_circ_0000231 overexpression (Fig. 8D). The value of C-caspase 3/t-caspase 3 was increased by Sev, but this result was reversed after upregulation of hsa_circ_0000231 (Fig. 8D), suggesting Sev could induce cell apoptosis by regulating hsa_circ_0000231. These data demonstrated that Sev repressed tumor growth by controlling hsa_circ_0000231 expression in vivo.

## Discussion

Emerging evidences suggest that anesthetics can influence cancer evolution [21]. Sev was found to serve as a cancer suppressor in CRC through various mechanisms. As reported by previous researchers, Sev restrained cell metastasis of CRC via modulating extracellular signal-regulated kinase/matrix metalloproteinase-9 signal path through increasing miR-203 [22]. Additionally, Sev suppressed cell metastasis via regulating miR-34a/ADAM metallopeptidase domain 10 (ADAM10) pathway in CRC [23]. In this paper, we found that Sev repressed CRC development. Different from the above results, Sev hindered CRC progression by regulating hsa_circ_0000231/miR-622 axis. In this research, to reveal the mechanism of Sev in mediating CRC process, the reasonable concentration of Sev was firstly determined. Thus, the impacts of various concentrations of Sev (0%, 1.7%, 3.4% and 5.1%) on CRC progression were explored. Results showed that Sev repressed cell viability, colony-forming ability and metastasis, and upregulated apoptosis rate as well as induced cell arrest in a concentration-dependent fashion. Also, Sev dose-dependently reduced the expression of c-Myc, K-RAS and BRAF. Based on these results, CRC cells were treated with 5.1% Sev in subsequent research.

Previous research revealed that circRNAs acted as tumor suppressors or promoters in CRC progression. For example, circ_001680 [24], circ_101555 [25] and circ_0053277 [26] contributed to CRC process, but circ_0009361 [27], circ_0020397 [28] and circ_0007534 [29] hindered CRC development via regulating cell proliferation, migration or apoptosis. In this paper, it was found that hsa_circ_0000231 content was increased in HCT116 and SW620 cells and the specimens of CRC patients, and its absence hindered CRC development by repressing cellular abilities in proliferation and metastasis as well as enhancing the capacity in apoptosis, which were approved by the findings of Liu et al. [30]. Beyond that, an inverse correlation between hsa_circ_0000231 level and the survival time of CRC sufferers was shown in this paper. Given the repressive impacts both Sev and the silencing of hsa_circ_0000231 on CRC progression, Sev-mediated effect on hsa_circ_0000231 expression was explored further. Data exhibited that hsa_circ_0000231 was dose-dependently downregulated by Sev. Thus, we suggested that hsa_circ_0000231 might be involved in Sev-mediated CRC progression. To elaborate on that, the overexpression plasmid of hsa_circ_0000231 was transfected into Sev-stimulated HCT116 and SW620 cells with control group, and same indices were detected. As expected, results exhibited that hsa_circ_0000231 overexpression attenuated Sev-mediated impacts on CRC process. Meanwhile, in vivo assay also showed enforced hsa_circ_0000231 expression restrained Sev-aroused inhibitive influences on tumor formation. The inhibition impact of Sev on PCNA protein expression and upregulation influence of that on the value of C-caspase 3/t-caspase 3 were also abolished after hsa_circ_0000231 overexpression in vivo. These evidences suggested that Sev could repress CRC development via modulating hsa_circ_0000231.

CircRNAs commonly interact with miRNAs in cancer progression [31]. Thus, the miRNA bound to hsa_circ_0000231 was assessed. Our results presented that hsa_circ_0000231 directly interacted with miR-622. As reported in previous research efforts, miR-622 acted as an anti-oncogene in cancer progression, such as gastric cancer [32] and renal cell carcinoma [33]. In CRC process, miR-622 could repress angiogenesis [34] and radiosensitivity [35]. In this study, a decreased expression of miR-622 was observed in CRC samples and cells, and miR-622 silencing promoted cell migration and invasion, which were supported by the current evidences [20, 36]. Besides, miR-622 inhibitors facilitated cellular proliferation and repressed cellular apoptosis. The impacts of miR-622 inhibitors on hsa_circ_0000231 absence-mediated CRC process also suggested that hsa_circ_0000231 modulated CRC development through sponging miR-622.

Additionally, our results supported that miR-622 expression was apparently upregulated after Sev treatment, which was reversed by enforced hsa_circ_0000231 expression. This result also suggested that Sev could increase miR-622 expression by regulating hsa_circ_0000231.

Collectively, ectopic hsa_circ_0000231 expression restrained Sev-mediated CRC progression. Besides, hsa_circ_0000231 bound to miR-622. MiR-622 inhibitors were further proved to hinder hsa_circ_0000231 absence-mediated repressive impacts on CRC process. Summarily, hsa_circ_0000231 restrained Sev-triggered repressive influences on CRC progression via binding to miR-622. This finding not only provides a theoretical foundation for further studying the application of Sev in CRC surgery, but also lays a basis for researching circRNA-directed CRC therapy.

## Supplementary Information

**Additional file 1: Figure S1.** The effects of Sev (0%, 1.7%, 3.4% and 5.1%) on the protein expression of c-Myc, K-RAS and BRAF were detected by western blot in both HCT116 (A) and SW620 cells (B). *p < 0.05 (ANOVA with Tukey’s test).

## Data Availability

Please contact the correspondence author for the data request.
