# Supplementary figures and images for: Sevoflurane inhibits malignant progression of colorectal cancer via hsa_circ_0000231-mediated miR-622
Source: J Biol Res (Thessalon). 2021 Jun 28;28:14. doi: 10.1186/s40709-021-00145-6 (PMC8237491; doi:10.1186/s40709-021-00145-6)

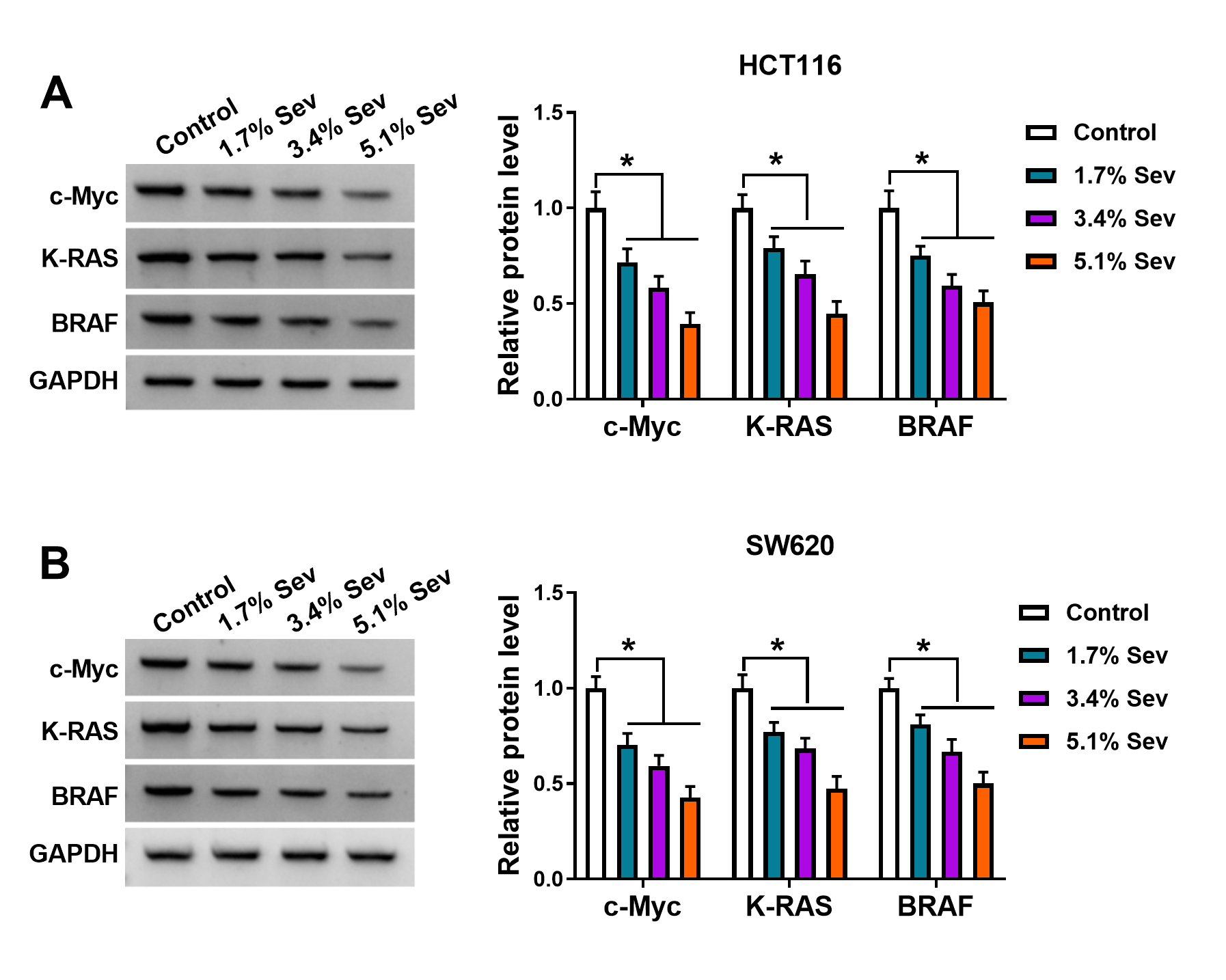

Supplement: Supplementary file 1 — Additional file 1: Figure S1. The effects of Sev (0%, 1.7%, 3.4% and 5.1%) on the protein expression of c-Myc, K-RAS and BRAF were detected by western blot in both HCT116 (A) and SW620 cells (B). *p < 0.05 (ANOVA with Tukey’s test). [file 40709_2021_145_MOESM1_ESM.tif]
